# North China Plain threatened by deadly heatwaves due to climate change and irrigation

**DOI:** 10.1038/s41467-018-05252-y

**Published:** 2018-07-31

**Authors:** Suchul Kang, Elfatih A. B. Eltahir

**Affiliations:** 1 Singapore-MIT Alliance for Research and Technology (SMART) Center for Environmental Sensing and Modeling (CENSAM), Singapore, 138602 Singapore; 2 Ralph M. Parsons Laboratory, Massachusetts Institute of Technology, Cambridge, MA 02139 USA

## Abstract

North China Plain is the heartland of modern China. This fertile plain has experienced vast expansion of irrigated agriculture which cools surface temperature and moistens surface air, but boosts integrated measures of temperature and humidity, and hence enhances intensity of heatwaves. Here, we project based on an ensemble of high-resolution regional climate model simulations that climate change would add significantly to the anthropogenic effects of irrigation, increasing the risk from heatwaves in this region. Under the business-as-usual scenario of greenhouse gas emissions, North China Plain is likely to experience deadly heatwaves with wet-bulb temperature exceeding the threshold defining what Chinese farmers may tolerate while working outdoors. China is currently the largest contributor to the emissions of greenhouse gases, with potentially serious implications to its own population: continuation of the current pattern of global emissions may limit habitability in the most populous region, of the most populous country on Earth.

## Abstract (web summary)

Irrigation increases the intensity of heatwaves over the North China Plain but how this will be exacerbated by climate change has not been quantified. Here the authors show that irrigation enhances magnitude of extreme wet-bulb temperature and intensity of heatwaves in this region.

## Introduction

**Fig. 1:**
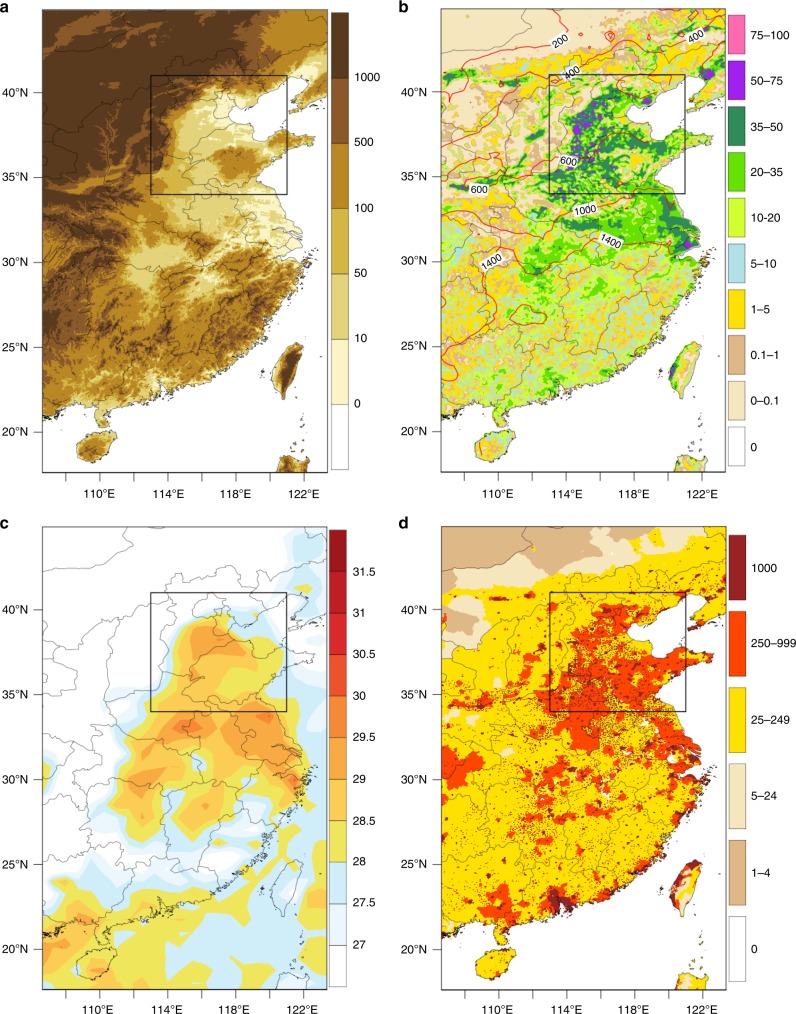
Brief characterization of Eastern China. Spatial distribution of **a** topographic map (m), **b** area equipped for irrigation (AEI, %) for 2005 from Historical Irrigation Data^40^ with climatology of annual precipitation from TRMM^49^ (contour, mm) in modern record (1998–2015), **c** highest daily maximum wet-bulb temperature from ERA-Interim^41^, TW_max_ (°C) in modern record (1979–2016), and **d** population density^53^ in people/km^2^. The box in each plot indicates the North China Plain used for regional analysis in this study. The figure was created using the NCAR Command Language (https://www.ncl.ucar.edu)

The North China Plain (NCP; defined here as 34°N to 41°N; 113°E to 121°E, see Fig. 1), with an area of about 400 thousand square kilometers, is the largest alluvial plain in China^1,2^. This region, inhabited by about 400 million, is one of the most densely populated in the world (Fig. 1). The rich soils in this region were formed by sedimentary deposits from the Yellow, Huai, and Hai rivers, providing excellent conditions for agriculture. The rainfall levels are relatively low compared to similar locations in South China, making irrigation necessary for supplementing soil water during the spring to early summer growing season (Fig. 1). This fertile plain has experienced vast expansion of irrigated agriculture which impacts significantly the surface radiation, surface energy balance, and boundary layer development in ways that impact surface humidity, and temperature. In observations that describe regional climate patterns, NCP stands out as a hot spot in the China-wide map of the maximum wet-bulb temperature (TW_max_) observed in the last few decades (Fig. 1c). Given these past observations, the region is expected to remain vulnerable to heatwaves in the future.

The frequency and intensity of heatwaves observed in China has increased significantly during the last 50 years as documented by several studies^3–6^. Severe heatwaves have been experienced in China, particularly since the beginning of the twenty-first century^5^. The surface mean temperature averaged over China has increased by about 1.35 °C during 1951–2006, a rate of about 0.24 °C/decade^7^, which is larger than the corresponding global rate of about 0.13 °C/decade during 1956–2005^8^. In July and August of 2003, extremely hot weather conditions lasting for 20–50 days occurred at many sites over South China^9^. During this period, the daily maximum temperature recorded in many locations south of Yangtze River was above 38 °C^10^. Heatwaves extended across most of China in 2006, with daily maximum temperature reaching 35 °C at 22 stations in Chongqing, Central China^11^. In summer 2013, a severe heatwave event occurred in Eastern China^12^. The daily maximum temperatures in many stations were extreme, breaking the historical records. Around Shanghai, the 141-year temperature record was exceeded, and the heatwave resulted in the death of dozens of people^13^.

Studies on heatwaves often (but not always) focus on surface temperature as the main variable used for characterizing their intensity^14,15^. However, consideration of surface humidity is as important as that of surface temperature in defining heatwaves, since humidity impacts how humans feel heat stress. Building on earlier work^15^ that defined a threshold for human survivability based on the magnitude of TW (an integrated measure of temperature and humidity), we have recently adopted the use of the daily TW_max_, computed from a 6-h moving average time series, for characterizing the intensity of heatwaves^16,17^. The choice of TW is motivated by the fact that the skin of a sweating human body can be approximated by this temperature, and the choice of 6 h is rooted in the assumption that a healthy human may not survive outdoors at a TW of 35 °C for more than 6 h. Hence, TW_max_ of 35 °C is assumed as physiologic threshold for survival of humans^18^. The choice of this variable has important implications regarding where we may expect to see severe heatwaves, since a typical TW (dry-bulb temperature) for a forest would be significantly warmer (colder) than the corresponding value for a desert along the same latitude.

Previous studies concluded that irrigation impacts the land-surface energy balance and atmospheric boundary layer development significantly^19,20^. The direct effects of irrigation are to enhance evapotranspiration, to cool the surface temperature, and to moisten the air^21,22^. The cooler surface emits significantly less long-wave radiation upward (Stephan–Boltzmann law), and the moistened air emits significantly more long-wave radiation towards the surface (water vapor greenhouse effect). As a result, irrigation tends to enhance the net long-wave radiation available to fuel total flux of latent and sensible heat into the atmosphere. Similarly, irrigation may impact how the surface absorbs incoming solar radiation^23^, but the sign of that effect would depend on the nature of land cover before irrigation. In addition, irrigation impacts the development of the atmospheric boundary layer^23^. Irrigation reduces the sensible heat flux resulting in lowering of the depth of the atmospheric boundary layer. Hence, irrigation enhances the total flux of heat only slightly, but results in a significantly shallower boundary layer. Considering the total energy of the surface air in the atmospheric boundary layer, including latent and sensible forms, as measured by the TW over extensively irrigated plains such as those in the NCP, we would expect an enhanced magnitude of the boundary layer energy per unit mass as measured by variables such as moist static energy or the TW.

In two recent studies^24,25^, the impact of climate change on heatwaves was investigated at the global scale, using observations and simulations of surface temperature and humidity. Although China was identified in one of these studies^25^ as one of the impacted regions, the relatively coarse resolution and global focus of their analysis precluded identification of hotspots for heatwaves at regional and local scales within China. The same studies did not include analysis of the impact of irrigation on heatwaves. The high resolution of our simulations, including representation of irrigation processes, makes it feasible to address these two important topics.

In this study, we project based on an ensemble of high-resolution regional climate model simulations that climate change would add significantly to the anthropogenic effects of irrigation, increasing the risk from heatwaves over the NCP region, an area with extensive irrigation development covering a relatively large fraction of the land surface. Under the business-as-usual (BAU) scenario of greenhouse gas emissions, NCP is likely to experience deadly heatwaves with TW exceeding the threshold defining what Chinese farmers may tolerate, while working outdoors without air conditioning.

## Results

### Model experiments and evaluation

Here, we use the Massachusetts Institute of Technology (MIT) Regional Climate Model (MRCM)^26^ with lateral boundary conditions obtained from simulations by a carefully selected set of global climate models from among those that participated in CMIP5^27^ (see selection criteria described in the Methods section and Supplementary Table 1 for the list of global climate models and their details). We perform simulations for historical period (1975–2005), as well as future climate (2070–2100) assuming two scenarios of GHG emissions^28^ (BAU scenario (RCP8.5) and moderate mitigation scenario (RCP4.5)) (see Supplementary Fig. 1, Supplementary Information for simulations domain, and Supplementary Table 2). For the historical and future period, we performed six sets of simulations, with and without irrigation. By comparing the historical period simulations, with and without irrigation, we estimate the impact of irrigation on heatwaves in the historical climate period as described by the TW. The results of these simulations reveal a significant role for irrigation in enhancing the magnitude of extreme TW_max_ and hence the intensity of heatwaves. (By “extreme” we mean maximum simulated value over this period.) Over the irrigated region and NCP, the extreme TW_max_ over a 30-year period increases by about 0.5 and 0.3 °C, respectively, as a result of irrigation during historical period. This impact of irrigation is even larger, if we consider the average TW_max_ instead of extreme values, and more pronounced during the relatively drier months of early summer (May and June) (see Supplementary Figs. 2–4). These are the months when irrigation has the largest impact on land surface conditions.

### Heatwaves due to climate change and irrigation

**Fig. 2:**
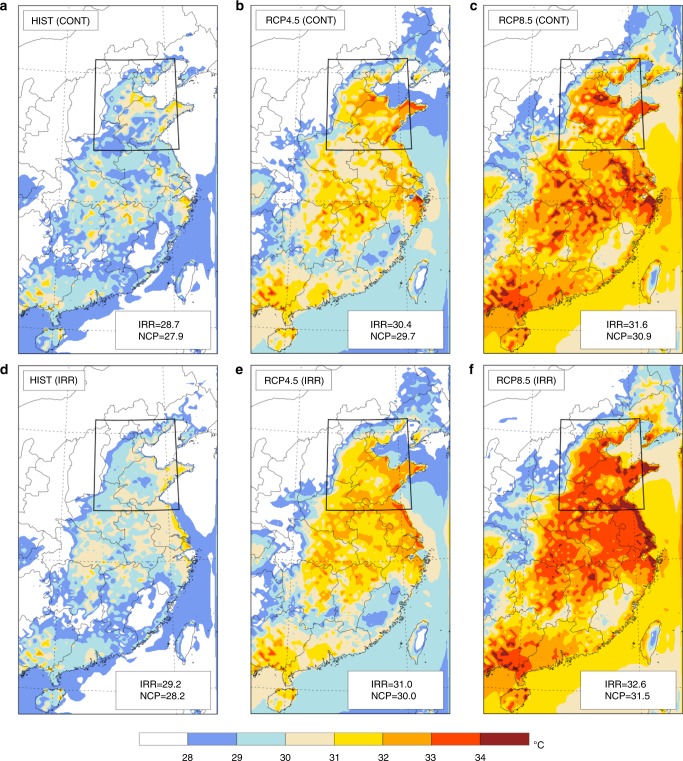
Spatial distribution of extreme wet-bulb temperature. Ensemble average of the 30-year maximum TW_max_ (°C) for irrigation activity and each GHG scenario: historical without irrigation activity (**a**), RCP4.5 without irrigation activity (**b**), RCP8.5 without irrigation activity (**c**), historical with irrigation activity (**d**), RCP4.5 with irrigation activity (**e**), and RCP8.5 with irrigation activity (**f**). Averages for irrigated region (IRR) and North China Plain (box in plot, NCP) are indicated in each plot. Extent of irrigated area is shown in Supplementary Fig. 1. TW_max_ is the maximum daily value from 6-h running average for each day (bias correction described in the Methods section). The figure was created using the NCAR Command Language (https://www.ncl.ucar.edu)

**Fig. 3:**
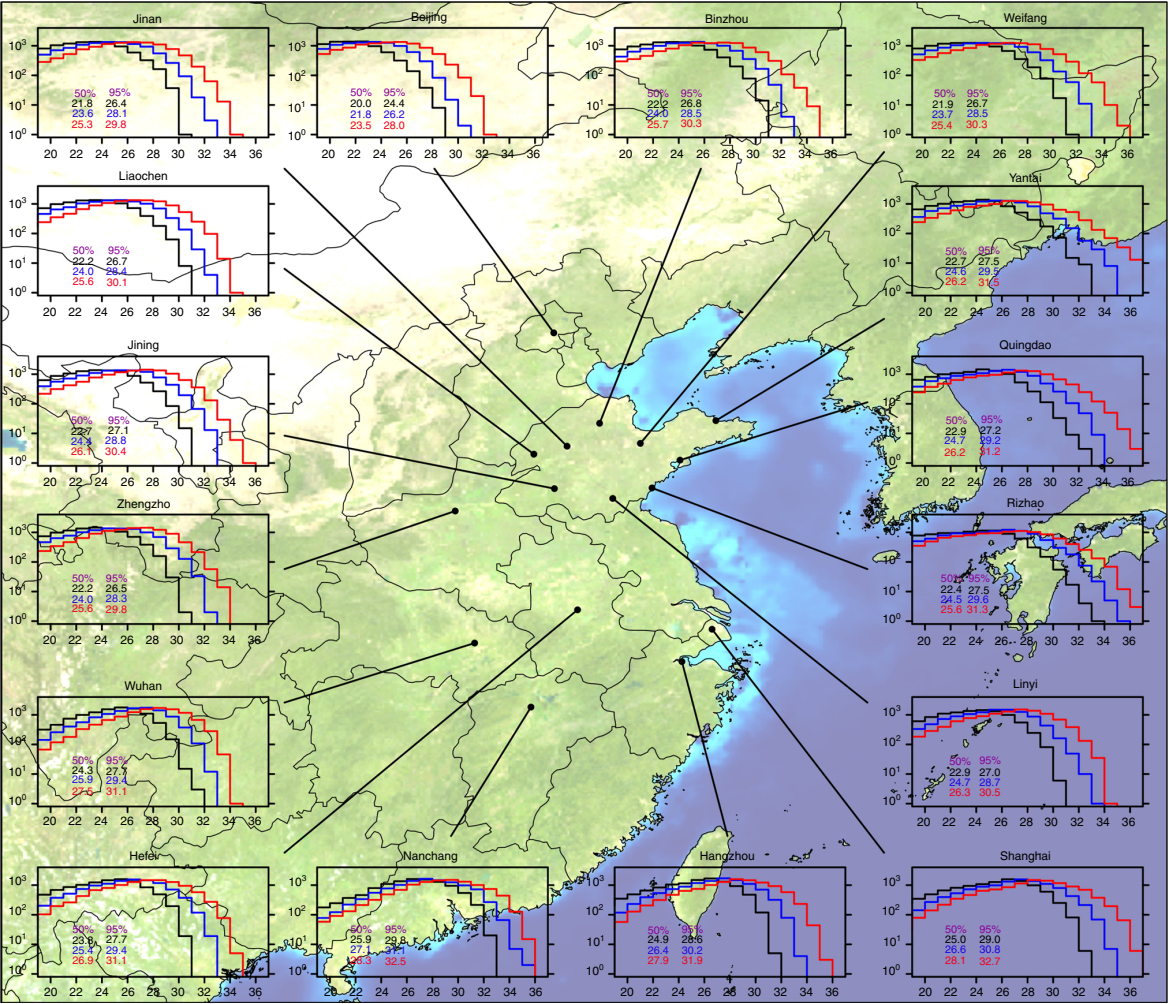
Histogram of daily maximum wet-bulb temperature in 16 cities over Eastern China. Histogram of the JJAS season of TW_max_ (°C) for each GHG scenario’s ensemble: historical (black), RCP4.5 (blue), and RCP8.5 (red). The histogram bin interval is 1.0 °C and the values on the *y*-axis indicate the number of exceedances. Values indicated within each plot represent the 50th and 95th percentile event thresholds. TW_max_ is the maximum daily value from 6-h running average for each day (bias correction described in the Methods section). The figure was created using the NCAR Command Language (https://www.ncl.ucar.edu), but the background image was obtained from NASA Visible Earth

The impacts of climate change are significantly larger than those of irrigation. The extreme TW_max_ over irrigated area and NCP are projected to increase by an additional 3.4 and 3.3 °C, respectively, assuming a BAU scenario of greenhouse gas (GHG) emissions with irrigation, while increases of extreme TW_max_ are 2.9 and 3 °C over irrigated area NCP, respectively, without irrigation (Fig. 2). The spatial distribution of TW_max_ under current and future climate features three regions with significantly warmer conditions: the NCP close to the Eastern coast, the Yangtze river valley, and the Southern coast. All these regions are characterized by relatively low elevation (lower than 50 m) compared to the surrounding area (e.g., Yan Mountain and Yaihang Mountain (Fig. 1a)), which is a major factor explaining occurrence of relatively warm conditions. Over several locations in the NCP and along the Eastern coast of China, such as the areas around Weifang, Jining, Qingdao, Rizhao, Yantai, Shanghai, and Hangzhou under the BAU scenario, TW_max_ exceeds the critical threshold for human survival of 35 °C, during several episodes over a 30-year period (Fig. 3). Moderate climate change mitigation efforts, represented by the RCP4.5 scenario of GHG emissions, reduce the risk of such heatwaves significantly; however, deadly heatwaves are still projected even under those conditions, though significantly less frequent (Fig. 3). In interpretation of the results of this study, we emphasize that TW_max_ values as low as 30 °C would qualify as “Extremely Dangerous” according to the National Oceanic and Atmospheric Administration (NOAA) Weather Service Heat Index (see Supplementary Table 6).

Irrigation enhances the response of maximum TW_max_ to climate change. The response of maximum TW_max_ over irrigated areas due to the combination of irrigation and climate change (estimated from Fig. 2a, f) is about 3.9 °C. This is larger than the sum of: (i) the response of maximum TW_max_ over irrigated areas to irrigation alone (estimated from Fig. 2a, d), of about 0.5 °C, and (ii) the response of maximum TW_max_ over the same areas (without irrigation) to climate change alone (estimated from Fig. 2a, c) of about 2.9 °C. This non-linearity in the response of TW to the combination of irrigation and climate change is likely caused by an enhanced water vapor feedback mechanism over irrigated areas. This topic will be investigated in our future research.

### Comparison of extreme heatwaves over NCP and Asian belt

**Fig. 4:**
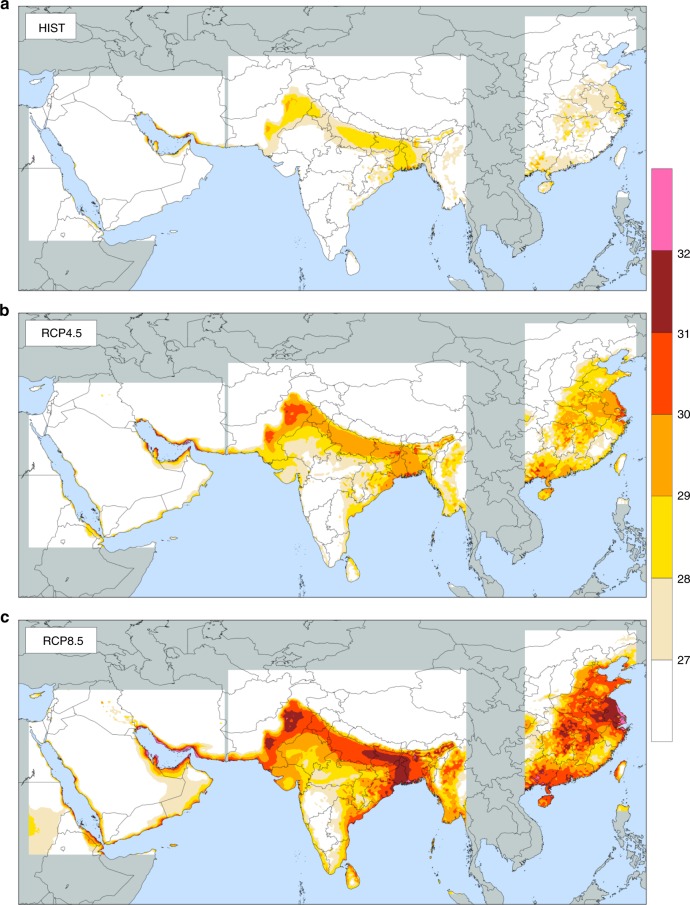
Spatial distribution of 95th percentile of daily maximum wet-bulb temperature. TW_max_ (°C) from the MRCM ensemble of simulations driven by three GCMs (Southwest Asia: CCSM, MPI, NorESM, South Asia, and Eastern China: CCSM, MPI, ACCESS) for each GHG scenario: historical (**a**), RCP4.5 (**b**), and RCP8.5 (**c**). All simulations include irrigation. TW_max_ is maximum daily value from 6-h running average for each day (bias correction described in the Methods setion). TW_max_ is presented over land areas only within the simulations domains. Land areas, outside simulations domains, are shown in gray. All ocean areas, within or outside simulations domains, are shown in blue. The figure was created using the NCAR Command Language (https://www.ncl.ucar.edu)

The impact of climate change over NCP is compared to the corresponding impacts in other locations within the Asian belt that was identified in our recent study^17^ as a hotspot for extreme heatwaves based on observations from the last few decades (Fig. 4). The response of extreme TW_max_ to the GHG forcing over NCP is significantly larger than the corresponding response over the Persian Gulf region or that over the Ganges and Indus valleys (see Supplementary Table 5). In comparison to the Persian Gulf and South Asia, NCP is the only region within that Asian belt that is not influenced by a warm ocean or sea next to it. This observation may explain some of the enhanced response in this region. If we use the 95% percentile value of TW_max_ as a measure of the intensity of extreme heatwaves, this variable increases by about 3 to 4 °C over the NCP, under RCP8.5, compared to an increase of 2 to 3 °C under the same scenario over the Persian Gulf region or the South Asia region (Supplementary Table 5). Taking this enhanced response to GHG forcing together with the simulated impact of irrigation in this region brings the extreme TW_max_ simulated by our model to approach and exceed the 35 °C threshold. NCP is likely to experience significantly warmer conditions and more severe heatwaves towards the end of the twenty-first century compared to the early decades of the twentieth century.

## Discussion

Although the model used in this study has been tested extensively over China and other regions of Asia, significant limitations may persist. The representation of surface processes and other physical parametrizations in our model represent a significant source of uncertainty. In order to minimize that source, we have tested the model against observations of surface temperature, humidity, and TW. As shown in the Supplementary Information (see Supplementary Table 4 and Supplementary Figs. 5–10), incorporation of irrigation improves significantly the skill of the model in reproducing the observed surface climate. Another source of uncertainty is the choice of the CMIP5 Global Climate Model simulations used in specifying the lateral boundary conditions for MRCM, historically and in the future. In order to reduce this uncertainty, we carefully selected only three climate models that show satisfactory performance in simulating the climate of this region (selection criteria described in the Methods section). Our results are based on the ensemble of simulations from three sets of experiments using the three different GCM simulations as boundary conditions.

This study complements our series of recent studies^16,17^ that documented a serious risk from potentially deadly heatwaves triggered by climate change in three discrete locations along an Asian belt stretching from the Persian Gulf to the Bohai Sea. Similar to our previous studies on the Persian Gulf and South Asia^16,17^, we emphasize the important role of humidity, and demonstrate a new approach to characterize heatwaves through the use of TW consistent with the physiologic threshold discussed above, and the use of 6-h period as the duration of a heatwave reflecting how long humans may survive at that threshold temperature. However, the magnitude of the projected enhancement of the risk of deadly heatwaves in China seems larger than the projected enhancements along the coasts of the Persian Gulf or over the Ganges and Indus valleys (Fig. 4).

China is currently the largest emitter of carbon dioxide, and the rates of emission has increased sharply since the beginning of the twenty-first century^29^. Our results suggest significant negative regional climate change impacts affecting the most populous region of China, NCP, where future population is projected to grow even further by the end of twenty-first century^30^. Although the emissions per capita from China may not stand out compared to other nations, national climate change policy in China will need to carefully evaluate and weigh the projected negative impacts of severe heatwaves on local population against the positive impacts of economic development. Enhanced investments in renewables, already adopted widely in China, may offer an alternative pathway that achieves economic development and mitigates local impacts of future global climate change. However, even under moderate mitigation scenarios severe heatwaves are projected for NCP which may necessitate simultaneous development of effective public health adaptation measures to avoid the deadly impacts of future heatwaves.

In this study, we project based on an ensemble of high-resolution regional climate model simulations that climate change would add significantly to the anthropogenic effects of irrigation, increasing the risk from heatwaves over the NCP region, an area with extensive irrigation development covering a relatively large fraction of the land surface. Under the BAU scenario of greenhouse gas emissions, NCP is likely to experience deadly heatwaves with TW exceeding the threshold defining what Chinese farmers may tolerate, while working outdoors without air conditioning.

## Methods

### Description of MRCM

MRCM^26^ used in the study is based on the Abdus Salam International Centre for Theoretical Physics Regional Climate Model Version 3 (RegCM3)^31^ but with several improvements^32–35^, achieved through incorporation of new physical schemes or modification of original schemes MRCM has been rigorously tested against observations, in its ability to simulate key observed climate features, across several regions (e.g., North America^32^, West Africa^36^, Southwest Asia^16^, South Asia^17^, Maritime Continent^37^). In particular, previous studies^38,39^ tested extensively the performance of the irrigation module used within MRCM. Hence, we use MRCM, including the irrigation module, to simulate climate over China, a region which probably has the largest irrigated area in the world^40^. Irrigation is simulated in the model, by replenishing the root-zone soil moisture to field capacity at the beginning of each month, whenever needed during summer (May to September), and wherever the grid point is equipped for irrigation according to the Historical Irrigation Data Set^40^.

### Simulation of the summer climate of North China Plain (NCP)

Before making climate projections, we analyze simulations by the MRCM constrained by boundary conditions from reanalysis data and evaluate its performance against observations. The rationale for carrying these experiments is to test the model skill in reproducing observed regional climate, as well as to study the impact of land use change on the historical climate of the region. As irrigation is widely practiced in NCP (Fig. 1b), it is essential that we evaluate the impact of irrigation on surface climate. In this regard, additional 30 years (1982–2011) numerical experiments consisting of control (CONT, without irrigation module) and irrigation (IRR, with irrigation module) simulations are performed using MRCM (Supplementary Table 3). The MRCM experiments adopt the same model configuration (e.g., domain coverage, spatiotemporal resolution, and physical parameters) as one used in the GCM downscaling, but are driven by the 1.5° × 1.5° 6-hourly ERA-Interim data^41^ as lateral boundary conditions and the 1° × 1° weekly NOAA optimum interpolation sea surface temperatures for the ocean surfaces^42^. To evaluate the two simulations, we compare the simulated surface conditions to the Climate Research Unit (CRU)^43^ data, focusing on several key surface variables such as surface temperature (Supplementary Figs. 5 and 6), specific humidity (Supplementary Figs. 7 and 8), and TW (Supplementary Figs. 9 and 10). The finger print of irrigation can be seen clearly by comparing field observations of surface conditions to the results of the numerical model simulations. Without explicit representation of irrigation and its direct and indirect effects, models would consistently simulate a summer climate over the NCP that features warmer temperature, drier air, and cooler TW. These difference between simulations and observations are consistent with the finger print of irrigation. Indeed, inclusion of a reasonable representation of irrigation into the model improves the correspondence between the model results and observations in terms of surface temperature, atmospheric humidity, and surface TW, leading to statistically significant reductions of overall biases. In a recent coordinated set of numerical models’ simulations over Eastern China, a warm and dry bias in surface conditions was identified as a common deficiency in the ability of other regional climate models (without irrigation) to simulate the observed climate of this region^44^.

### Regional climate change projections

The model domain covers Eastern China including NCP, which is centered at 115°E and 31.5°N with a 25 km grid spacing on a Lambert conformal projection (Supplementary Fig. 1). The atmospheric lateral boundary conditions for MRCM are specified based on GCM simulations, selected from among CMIP5 ^27^ participant models. Since the regional climate model is constrained by atmospheric lateral boundary conditions, selection of the GCMs is important. In this study, three GCMs are carefully selected based on a screening process, including rigorous evaluation of the GCM performance in simulating key climate variables for the historical period over the target domain (see selection of GCMs section below). The selected models are the Community Climate System Model Version 4 (hereafter referred to as CCSM)^45^, Australian Community Climate and Earth System Simulator Version 1.0 (hereafter referred to as ACCESS)^46^, and Max-Planck-Institution Earth System Model running on Medium Resolution grid (hereafter referred to as MPI)^47^. For each selected GCM, two historical climate simulations are performed assuming historical GHG concentrations, with and without irrigation module (HIST), for the period 1975–2005. To quantify the impact of a range of GHG concentrations, four future projection simulations, with and without irrigation module, are performed with two different RCP scenarios^28^, namely, RCP4.5 and RCP8.5 for the period 2070–2100 (Supplementary Table 2). RCP8.5 is a rising concentration pathway leading to 8.5 W m^−2^ of radiative forcing by 2100 and can be considered a BAU scenario. RCP4.5 is a stabilization scenario after about 2060, leading to 4.5 W m^−2^ of radiative forcing by 2100. It represents moderate mitigation effort. In total, six sets of experiments are performed with MRCM over Eastern China. Each set of experiments consists of three ensemble members forced by three GCMs. The historical baseline period consists of 31 years from 1975 to 2005 and the projected future period covers 31 years from 2070 to 2100.

### Selection of the GCMs

The GCMs used for specifying the boundary conditions for MRCM are selected from among the many participant models in CMIP5 by applying the following criteria:

We adopt the 19 GCMs evaluated favorably by McSweeney et al.^48^ based on their performance over Southeast Asia. And then we select 6 GCMs out of the 19 by requiring an oceanic horizontal resolution of 1.11° or higher, capable of simulating complex ocean processes over this region. Over land, the surface temperature, TW, relative humidity, and precipitation from GCMs are objectively analyzed and compared to CRU^43^, ERA-Interim^41^, and TRMM^49^ datasets. To assess the performance of each GCM, the NRMSE, PCC, and annual cycle for each variable are compared separately over two regions (Northeastern China and Southeastern China).

As a result of applying the above criteria, three GCMs are selected: CCSM4^45^, ACCESS1.0 ^46^, and MPI-ESM-MR^47^. More detailed information about the GCMs is presented in Supplementary Table 1.

### Bias correction

Simulations by a regional climate model may contain a systematic bias arising from inadequate physics, and/or bias in the global climate model simulations used as lateral boundary conditions^50,51^. These model biases impact historical as well as future climate change projections. To reduce the impact of this bias, we applied the same bias correction procedure developed by Pal and Eltahir^16^ for correcting future projections in southwest Asia. This methodology allows correction of daily TW_max_. TW is computed by the formulation developed by Davis-Jones^52^. Reliable reanalysis data at high spatial and temporal resolution is the key to correct bias in simulated daily variables. ERA-Interim reanalysis represents spatially complete and dynamically consistent estimates of the state of the climate system^41^ and is therefore used for the following bias correction procedure. In the first step, TW is computed by the formulation developed by Davies-Jones^52^for both the MRCM hourly output and the ERA-Interim reanalysis 3-hourly 0.75° × 0.75° data. In this stage, the 6-h running average (6-h window) is calculated and then its daily maximum (denoted by TW_max_) is selected for each day, and then the ERA-Interim TW_max_ data are transferred from the 0.75° × 0.75° horizontal grid to the 25-km MRCM grid. In the second step, consistent MRCM and ERA-Interim climatologies of TW_max_ is computed for each day of the year on the MRCM 25-km grid. In the final step, the magnitude of the bias for each day of the year is estimated by the difference between 30-day running means of the two climatologies. The daily bias is then applied to the MRCM daily values of TW_max_ for the present-day and future climates.

### Code availability

Code from this study is available from the corresponding author upon reasonable request.

### Data availability

The Global 30 Arc-Second Elevation (GTOPO30) used in Fig. 1a is from International Centre for Theoretical Physics (ICTP) data server (http://clima-dods.ictp.it/data/regcm4/SURFACE). The area equipped for irrigation (AEI) used in Fig. 1b is from Historical Irrigation Data (https://mygeohub.org/publications/8/2). The annual precipitation used in Fig. 1b is from Tropical Rainfall Measuring Mission (TRMM, https://trmm.gsfc.nasa.gov/). The 3-hourly temperature and dew point temperature to calculate daily TW_max_ used in Fig. 1c are from the ERA-Interim reanalysis (http://apps.ecmwf.int/datasets/). The population density used in Fig. 1d is from Global Rural–Urban Mapping Project (GRUMP, http://sedac.ciesin.columbia.edu/data/set/grump-v1-population-density).

All MIT Regional Climate Model results used for the present study are available from the authors on reasonable request.

## Electronic supplementary material

Supplementary Information

## References

[1] Liu J, Zheng C, Zheng L, Lei Y (2008). Ground water sustainability: methodology and application to the North China Plain. Ground Water.

[2] Zheng C (2010). Can China cope with its water crisis?—perspectives from the North China Plain. Ground Water.

[3] Wang XL, Gaffen DJ (2001). Trends in extremes of surface humidity, temperature, and summertime heat stress in China. Adv. Atmos. Sci..

[4] Ding T, Qian W (2011). Geographical patterns and temporal variations of regional dry and wet heatwave events in China during 1960–2008. Adv. Atmos. Sci..

[5] Sun Y (2014). Rapid increase in the risk of extreme summer heat in Eastern China. Nat. Clim. Change.

[6] Wu, J., Gao, X., Giorgi, F. & Chen, D. Changes of effective temperature and cold/hot days in late decades over China based on a high resolution gridded observation dataset. *Int. J. Climatol*. **37**, 788–800 (2017).

[7] Piao SL (2010). The impacts of climate change on water resources and agriculture in China. Nature.

[8] IPCC. *Climate Change 2007: The Physical Science Basis*(eds Solomon, S. et al.) (Cambridge University Press, Cambridge, 2007).

[9] Wang YW, Zhai PM, Tian H (2006). Extreme high temperatures in Southern China in 2003 under the background of climate change. Meteorol. Mon..

[10] Xu B, Xu AH, Tang CS (2003). Severe high temperature analysis in Jiangxi during summer 2003. Jiangxi Meteorol. Sci. Technol..

[11] Chen HB, Fan XH (2007). Some extreme events of weather, climate and related phenomena in 2006. Clim. Environ. Res..

[12] Hou W (2014). Climatic characteristics over China in 2013. Meteorol. Mon..

[13] Sun X (2014). Effects of temperature and heat waves on emergency department visits and emergency ambulance dispatches in Pudong new area, China: a time series analysis. Environ. Health.

[14] Meehl GA, Tebaldi C (2004). More intense, more frequent, and longer lasting heatwaves in the 21st century. Science.

[15] Anderson GB, Bell ML (2011). Heat waves in the United States: mortality risk during heat waves and effect modification by heat wave characteristics in 43 U.S. communities. Environ. Health Perspect..

[16] Pal JS, Eltahir EAB (2016). Future temperature in southwest Asia projected to exceed a threshold for human adaptability. Nat. Clim. Change.

[17] Im ES, Pal JS, Eltahir EAB (2017). Deadly heat waves projected in the densely populated agricultural regions of South Asia. Sci. Adv..

[18] Sherwood SC, Huber M (2010). An adaptability limit to climate change due to heat stress. Proc. Natl. Acad. Sci. USA.

[19] Douglas EM (2009). The impact of agricultural intensification and irrigation on land–atmosphere interactions and Indian monsoon precipitation—a mesoscale modeling perspective. Glob. Planet. Change.

[20] Qian Y, Huang M, Yang B, Berg LK (2013). A modeling study of irrigation effects on surface fluxes and land–air–cloud interactions in the Southern Great Plains. J. Hydrometeorol..

[21] Kueppers LM, Snyder MA, Sloan LC (2007). Irrigation cooling effect: regional climate forcing by land-use change. Geophys. Res. Lett..

[22] Sacks W, Cook B, Buenning N, Levis S, Helkowski J (2009). Effects of global irrigation on the near-surface climate. Clim. Dyn..

[23] Im ES, Eltahir EAB (2014). Enhancement of rainfall and runoff upstream from irrigation location in a climate model of West Africa. Water Resour. Res..

[24] Mora C (2017). Global risk of deadly heat. Nat. Clim. Change.

[25] Russo S, Sillmann J, Sterl A (2017). Humid heat waves at different warming levels. Sci. Rep..

[26] Im ES, Gianotti RL, Eltahir EAB (2014). Improving the simulation of the West African monsoon using the MIT Regional Climate Model. J. Clim..

[27] Taylor KE, Stouffer RJ, Meehl GA (2012). An overview of CMIP5 and the experiment design. Bull. Am. Meteorol. Soc..

[28] Van Vuuren D (2011). The representative concentration pathways: an overview. Clim. Change.

[29] Le Quéré C (2016). Global carbon budget 2016. Earth Syst. Sci. Data.

[30] Shi Y, Gao XJ, Xu Y, Giorgi F, Chen DL (2016). Effects of climate change on heating and cooling degree days and potential energy demand in the household sector of China. Clim. Res..

[31] Pal JS (2007). Regional climate modeling for the developing world: the ICTP RegCM3 and RegCNET. Bull. Am. Meteorol. Soc..

[32] Winter JM, Pal JS, Eltahir EAB (2009). Coupling of integrated biosphere simulator to Regional Climate Model Version 3. J. Clim..

[33] Gianotti RL, Eltahir EAB (2014). Regional climate modeling over the maritime continent. Part I: New parameterization for convective cloud fraction. J. Clim..

[34] Gianotti RL, Eltahir EAB (2014). Regional climate modeling over the maritime continent. Part II: New parameterization for autoconversion of convective rainfall. J. Clim..

[35] Gianotti RL, Zhang D, Eltahir EAB (2012). Assessment of the Regional Climate Model Version 3 over the Maritime Continent using different cumulus parameterization and land surface schemes. J. Clim..

[36] Im, E.-S. & Eltahir, E. A. B. Simulations of the observed ‘jump’ in the West African monsoon and its underlying dynamics using the MIT regional climate model. *Int. J. Climatol*. **38**, 841–852 (2018).

[37] Im, E.-S. & Eltahir, E. A. B. Simulation of the diurnal variation of rainfall over the western Maritime Continent using a regional climate model. *Clim. Dyn*. **51**, 73–88 (2018).

[38] Im ES, Marcella MP, Eltahir EAB (2014). Impact of potential large-scale irrigation on the West African Monsoon and its dependence on location of irrigated area. J. Clim..

[39] Marcella MP, Eltahir EAB (2014). Introducing an irrigation scheme to a regional climate model: a case study over West Africa. J. Clim..

[40] Siebert S (2015). A global data set of the extent of irrigated land from 1900 to 2005. Hydrol. Earth Syst. Sci..

[41] Reynolds RW (2002). An improved in situ and satellite SST analysis for climate. J. Clim..

[42] Harris I, Jones PD, Osborn TJ, Lister DH (2014). Updated high-resolution grids of monthly climatic observations—the CRU TS3.10 dataset. Int. J. Climatol..

[43] Park C (2016). Evaluation of multiple regional climate models for summer climate extremes over East Asia. Clim. Dyn..

[44] Meehl GA (2012). Climate system response to external forcings and climate change projections in CCSM4. J. Clim..

[45] Bi D (2013). The ACCESS coupled model: description, control climate and evaluation. Austr. Meteorol. Oceanogr. J..

[46] Giorgetta MA (2013). Climate and carbon cycle changes from 1850 to 2100 in MPI-ESM simulations for the Coupled Model Intercomparison Project phase 5. J. Adv. Model. Earth Syst..

[47] McSweeney CF, Jones RG, Lee RW, Rowell DP (2015). Selecting CMIP5 GCMs for downscaling over multiple regions. Clim. Dyn..

[48] Balk DL (2006). Determining global population distribution: methods, applications and data. Adv. Parasitol..

[49] Dee DP (2011). The ERA-Interim reanalysis: configuration and performance of the data assimilation system. Q. J. R. Meteorol. Soc..

[50] Liang X (2008). Regional climate models downscaling analysis of general circulation models present climate biases propagation into future change projections. Geophys. Res. Lett..

[51] Ehret U (2012). Should we apply bias correction to global and regional climate model data?. Hydrol. Earth Syst. Sci. Discuss..

[52] Davies-Jones R (2008). An efficient and accurate method for computing the wet-bulb temperature along pseudoadiabats. Mon. Weath. Rev..

[53] Huffman GJ (2007). The TRMM Multi-satellite Precipitation Analysis (TMPA): quasi-global, multiyear, combined-sensor precipitation estimates at fine scales. J. Hydrometeorol..

