# Supplementary material for: North China Plain threatened by deadly heatwaves due to climate change and irrigation
Source: Nat Commun. 2018 Jul 31;9:2894. doi: 10.1038/s41467-018-05252-y (PMC6068174; doi:10.1038/s41467-018-05252-y)
Supplement: Supplementary file 1 — Supplementary Information [file 41467_2018_5252_MOESM1_ESM.pdf]

North China Plain threatened by deadly heatwaves due to climate change and irrigation

Suchul KANG & Elfatih A B ELTAHIR

Supplementary Information

Supplementary Table 1. Descriptions of Global Climate Models used in this study.

Model	Atmosphere (latitude x longitude)	Ocean (latitude x longitude)	Institute	Main reference
Community Climate System Model Version 4 (CCSM4)	0.9^o x 1.25^o	1.11^o x 0.27^o ~0.54^o	National Center for Atmospheric Research (NCAR)	Gent et al. 2011
Max-Planck-Institute Earth System Model Medium resolution (MPI-ESM-MR)	T63(˜1.875^o)	0.4^o x 0.4^o	Max Planck Institute for Meteorology (MPI)	Giorgetta et al. 2013
Australian Community Climate and Earth System Simulator Version 1.0 (ACCESS 1.0)	1.25^o x 1.875^o	$1/3^o$ ~ 1°	Commonwealth Scientific and Industrial Research Organization (CSIRO) and Bureau of Meteorology (BMC)	Bi et al. 2013

Supplementary Table 2. Summary of twelve MRCM simulations, forced by three GCMs for each GHG scenario.

Boundary condition	HIST(CONT)	HIST(IRR)	RCP4.5(IRR)	RCP 8.5(IRR)
**MPI**	- Historical period (1975-2005)<br>- Simulation without irrigation module	- Historical period (1975-2005)<br>- Simulation with irrigation module	- Future period (2070-2100)<br>- Simulation with irrigation module	- Future period (2070-2100)<br>- Simulation with irrigation module
**ACCESS**	- Historical period (1975-2005)<br>- Simulation without irrigation module	- Historical period (1975-2005)<br>- Simulation with irrigation module	- Future period (2070-2100)<br>- Simulation with irrigation module	- Future period (2070-2100)<br>- Simulation with irrigation module
**CCSM**	- Historical period (1975-2005)<br>- Simulation without irrigation module	- Historical period (1975-2005)<br>- Simulation with irrigation module	- Future period (2070-2100)<br>- Simulation with irrigation module	- Future period (2070-2100)<br>- Simulation with irrigation module

Supplementary Table 3. Summary of two MRCM simulations forced by ERA-interim data.

Boundary condition	CONT	IRR
**ERA-interim**	- Simulation period (1982-2011)<br>- Simulation without irrigation module	- Simulation period (1982-2011)<br>- Simulation with irrigation module

Supplementary Table 4. May-June-July (MJJ) and July-August-September (JAS) 30-year climatology of surface radiation (unit: Wm^{-2}), heat flux components (unit: Wm^{-2}) and PBL height (unit: m) averaged over irrigated area (land cover is 13 in Fig S7) derived from MRCM forced by Era-interim data without irrigation module (CONT) and one with irrigation module (IRR).

Season	Variables	CONT	IRR
MJJ	Absorbed shortwave radiation	221	201
	Downward longwave radiation	301	314
	Upward longwave radiation	385	373
	Sensible heat flux	80	14
	Latent heat flux	53	122
	Sensible heat flux + Latent heat flux	133	136
	PBL height	865	482
JAS	Absorbed shortwave radiation	203	183
	Downward longwave radiation	313	323
	Upward longwave radiation	391	379
	Sensible heat flux	57	46
	Latent heat flux	64	77
	Sensible heat flux + Latent heat flux	121	123
	PBL height	793	442

Supplementary Table 5. 95th percentile of TW_{max} for Historical period and their changes in RCP 4.5 and RCP 8.5 at cities.

		**HIST**	**RCP 4.5**	**RCP 8.5**
**Southwest Asia**	Dhahran, Saudi Arabia	29.4	+ 1.4	+ 2.9
	Doha, Qatar	28.7	+ 1.5	+ 2.9
	Abu Dhabi, UAE	28.3	+ 1.1	+ 2.4
	Dubai, UAE	28.8	+ 1.2	+ 2.7
**South Asia**	Lahore, Pakistan	27.9	+ 1.5	+ 2.4
	Lucknow, India	28.3	+ 1.4	+ 2.5
	Patna, India	28.5	+ 1.3	+ 2.7
	Dhaka Bangladesh	28.0	+ 1.4	+ 2.7
**Eastern China**	Shanghai, China	29.0	+ 1.8	+ 3.7
	Hangzhou, China	28.3	+ 1.7	+ 3.4
	Qingdao, China	27.2	+ 2.1	+ 3.9
	Rizhao, China	27.5	+ 2.1	+ 3.8

Supplementary Table 6. Table of *TW* (upper) and NOAA National Weather Service Heat Index (lower) generated from the same temperature and humidity.

Wet-Bulb Temperature

Tempearature

Relative Humidity (%)

°C	26.7	27.8	28.9	30.0	31.1	32.2	33.3	34.4	35.6	36.7	37.8	38.9	40.0	41.1	42.2	43.3	44.4	45.6	46.7	47.8	48.9	50.0	51.1	52.2	53.3	54.4
°F	80	82	84	86	88	90	92	94	96	98	100	102	104	106	108	110	112	114	116	118	120	122	124	126	128	130
40	17.0	17.8	18.7	19.6	20.4	21.3	22.1	22.9	23.8	24.6	25.5	26.4	27.3	28.2	29.1	30.0	30.9	31.8	32.7	33.5	34.4	35.3	36.2	37.1	38.0	38.9
45	17.9	18.8	19.7	20.6	21.4	22.3	23.2	24.1	25.0	25.9	26.8	27.7	28.7	29.6	30.5	31.4	32.3	33.2	34.2	35.1	36.0	36.9	37.8	38.7	39.7	40.6
50	18.8	19.7	20.6	21.5	22.4	23.3	24.2	25.2	26.1	27.1	28.0	29.0	29.9	30.9	31.8	32.8	33.7	34.6	35.6	36.5	37.5	38.4	39.4	40.3	41.3	42.4
55	19.7	20.6	21.5	22.5	23.4	24.3	25.3	26.3	27.2	28.2	29.2	30.2	31.1	32.1	33.1	34.0	35.0	35.9	36.9	37.9	38.9	39.8	40.9	41.9	43.0	44.1
60	20.5	21.4	22.4	23.3	24.3	25.3	26.3	27.3	28.3	29.3	30.3	31.3	32.3	33.2	34.2	35.2	36.2	37.2	38.2	39.2	40.2	41.2	42.3	43.4	44.6	45.9
65	21.3	22.3	23.2	24.2	25.2	26.2	27.3	28.3	29.3	30.3	31.3	32.3	33.3	34.3	35.3	36.4	37.4	38.4	39.4	40.4	41.5	42.6	43.8	45.0	46.3	47.7
70	22.1	23.0	24.0	25.1	26.1	27.2	28.2	29.2	30.3	31.3	32.3	33.4	34.4	35.4	36.4	37.4	38.5	39.5	40.6	41.7	42.8	44.0	45.3	46.6	48.1	49.7
75	22.8	23.8	24.8	25.9	27.0	28.0	29.1	30.1	31.2	32.2	33.3	34.3	35.4	36.4	37.5	38.5	39.6	40.7	41.8	42.9	44.1	45.4	46.8	48.3	50.0	51.9
80	23.5	24.6	25.6	26.7	27.8	28.9	30.0	31.0	32.1	33.1	34.2	35.3	36.3	37.4	38.4	39.5	40.6	41.8	42.9	44.2	45.5	46.9	48.4	50.1	52.1	54.4
85	24.2	25.3	26.4	27.5	28.6	29.7	30.8	31.9	32.9	34.0	35.1	36.2	37.2	38.3	39.4	40.5	41.7	42.9	44.1	45.4	46.8	48.4	50.2	52.2	54.5	57.3
90	24.9	26.0	27.2	28.3	29.4	30.5	31.6	32.7	33.8	34.9	35.9	37.0	38.1	39.2	40.4	41.5	42.7	44.0	45.3	46.7	48.3	50.0	52.1	54.4	57.2	60.6
95	25.6	26.7	27.9	29.0	30.1	31.2	32.4	33.5	34.6	35.7	36.8	37.9	39.0	40.1	41.3	42.5	43.7	45.1	46.5	48.1	49.8	51.8	54.2	56.9	60.3	64.5
100	26.3	27.4	28.6	29.7	30.9	32.0	33.1	34.2	35.3	36.5	37.6	38.7	39.8	41.0	42.2	43.5	44.8	46.2	47.8	49.5	51.5	53.8	56.5	59.8	64.0	69.2

Caution Extreme Caution Danger Extreme Danger

NOAA National Weather Service Heat Index

Temperature

Relative Humidity (%)

°C	26.7	27.8	28.9	30.0	31.1	32.2	33.3	34.4	35.6	36.7	37.8	38.9	40.0	41.1	42.2	43.3	44.4	45.6	46.7	47.8	48.9	50.0	51.1	52.2	53.3	54.4
°F	80	82	84	86	88	90	92	94	96	98	100	102	104	106	108	110	112	114	116	118	120	122	124	126	128	130
40	80	81	83	85	88	91	94	97	101	105	109	114	119	124	130	136	142	148	155	162	170	178	186	194	203	212
45	80	82	84	87	89	92	96	100	104	109	114	119	124	130	137	143	150	158	166	174	182	191	200	210	220	230
50	81	83	85	88	91	95	99	103	108	113	118	124	131	137	144	152	160	168	177	186	196	205	216	226	238	249
55	81	84	86	89	93	97	101	106	112	117	124	130	137	145	153	161	170	179	189	199	210	221	232	244	256	269
60	82	84	88	91	95	100	105	110	116	123	129	137	145	153	162	171	181	191	202	213	225	237	250	263	277	291
65	82	85	89	93	98	103	108	114	121	128	136	144	153	162	172	182	193	204	216	228	241	254	268	283	298	313
70	83	86	90	95	100	106	112	119	126	134	143	152	161	172	182	194	205	218	231	244	258	273	288	304	320	337
75	84	88	92	97	103	109	116	124	132	141	150	160	171	182	193	206	219	232	246	261	276	292	308	325	343	361
80	84	89	94	100	106	113	121	129	138	148	158	169	181	193	205	219	233	247	263	279	295	312	330	348	368	387
85	85	90	96	102	110	117	126	135	145	155	167	179	191	204	218	233	248	264	280	297	315	333	353	372	393	414
90	86	91	98	105	113	122	131	141	152	164	176	189	202	216	231	247	263	280	298	317	336	356	376	398	420	442
95	86	93	100	108	117	127	137	148	160	172	185	199	214	229	245	262	280	298	317	337	358	379	401	424	447	471
100	87	95	103	112	121	132	143	155	168	181	195	210	226	243	260	278	297	317	337	358	380	403	427	451	476	502

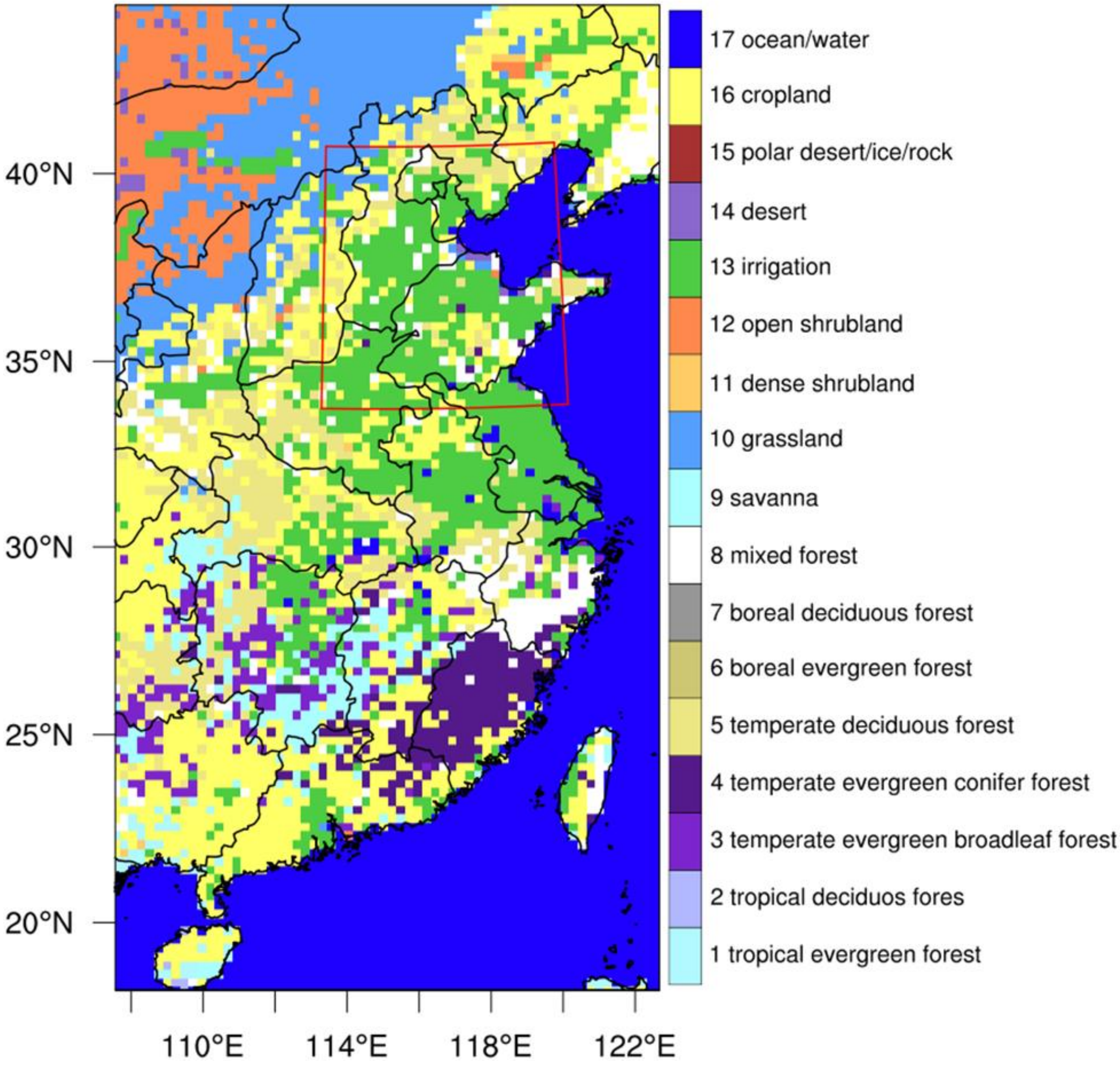

Supplementary Figure 1. Map of the 25km-resolution MRCM domain including land cover. The MRCM grid, centered at 31.5ºN and 115ºE on a Lambert Conformal projection, consists of 70 points in the x-direction and 121 points in the y-direction. The box indicates the North China Plain used for regional analysis in this study.). The figure was created using the NCAR Command Language (https://www.ncl.ucar.edu)

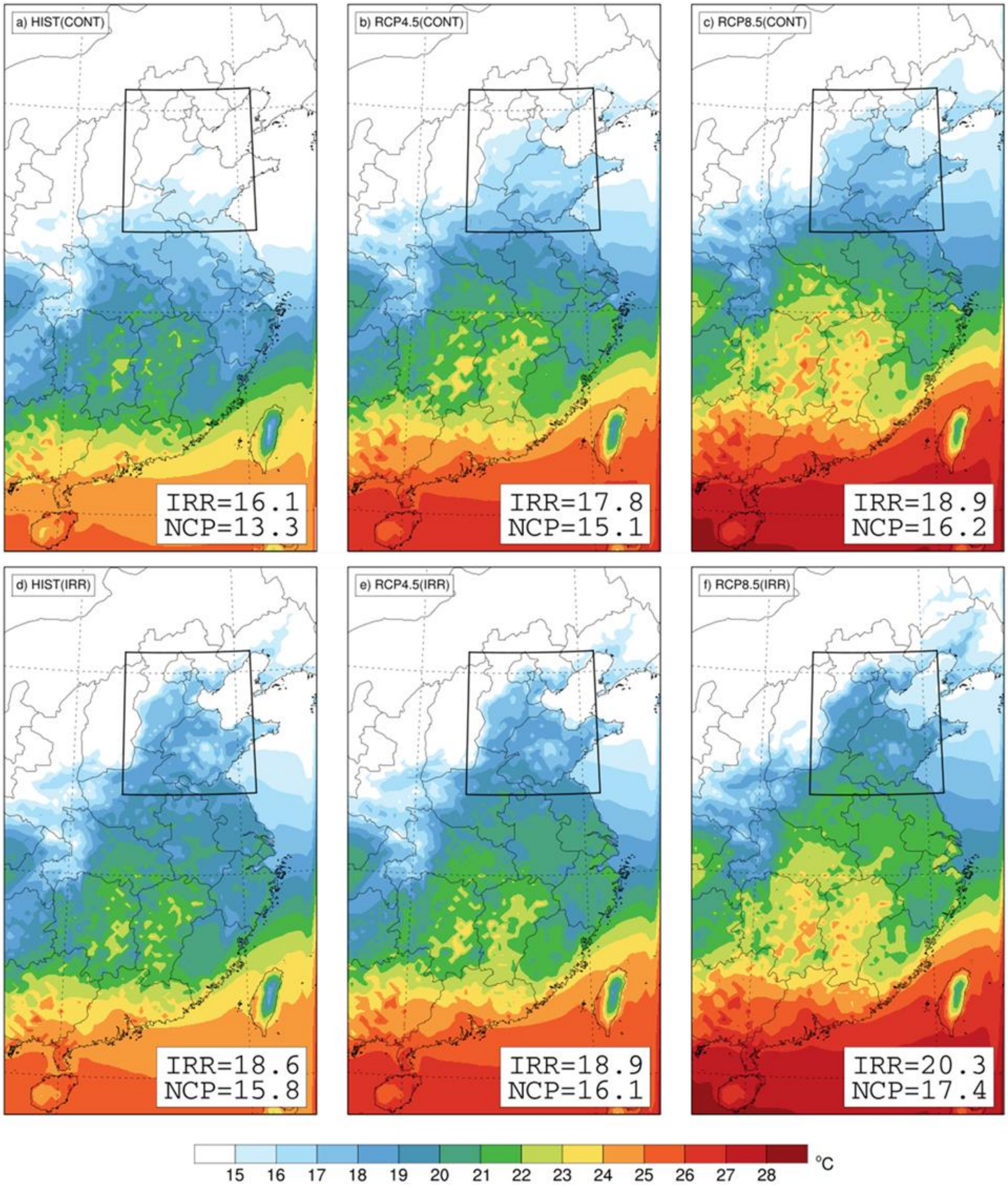

Supplementary Figure 2. Spatial distribution of ensemble mean of May-June 30-year climatology of TWmax (°C) for irrigation activity and each GHG scenario: historical without irrigation activity (a), RCP 4.5 without irrigation activity (b), RCP8.5 without irrigation activity (c), historical with irrigation activity (d), RCP 4.5 with irrigation activity (e) and RCP8.5 with irrigation activity (f). Averages for irrigated region (IRR) and North China Plain (box in plot, NCP) are indicated in each plot. Extent of irrigated area is shown in Supplementary Figure 1. TWmax is maximum daily value from 6-hour running average for each day (bias correction described in Method). The figure was created using the NCAR Command Language (https://www.ncl.ucar.edu)

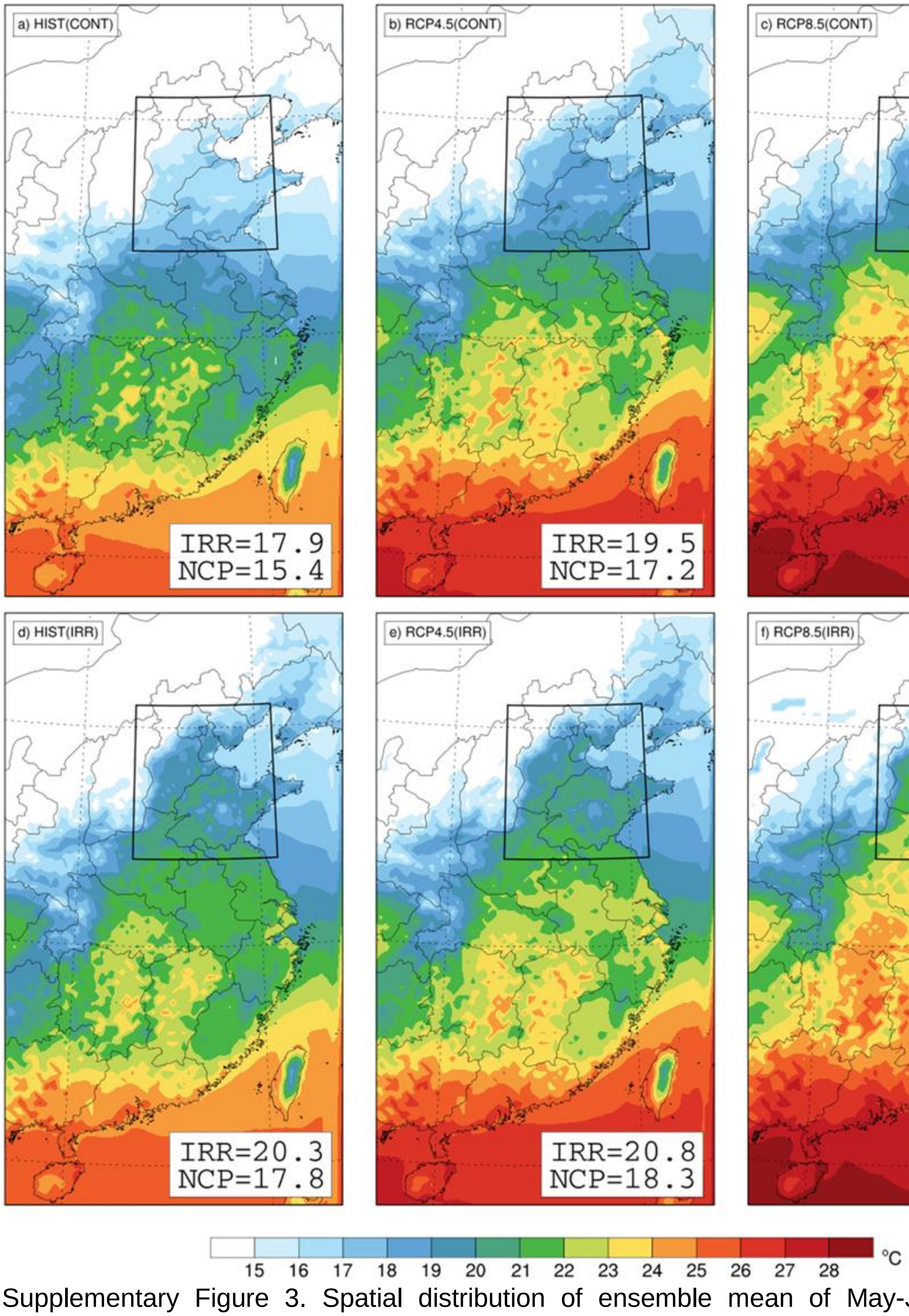

Supplementary Figure 3. Spatial distribution of ensemble mean of May-June-July 30-year climatology of TWmax (°C) for irrigation activity and each GHG scenario: historical without irrigation activity (a), RCP 4.5 without irrigation activity (b), RCP8.5 without irrigation activity (c), historical with irrigation activity (d), RCP 4.5 with irrigation activity (e) and RCP8.5 with irrigation activity (f). Averages for irrigated region (IRR) and North China Plain (box in plot, NCP) are indicated in each plot. Extent of irrigated area is shown in Supplementary Figure 1. TWmax is maximum daily value from 6-hour running average for each day (bias correction described in Method). The figure was created using the NCAR Command Language (https://www.ncl.ucar.edu)

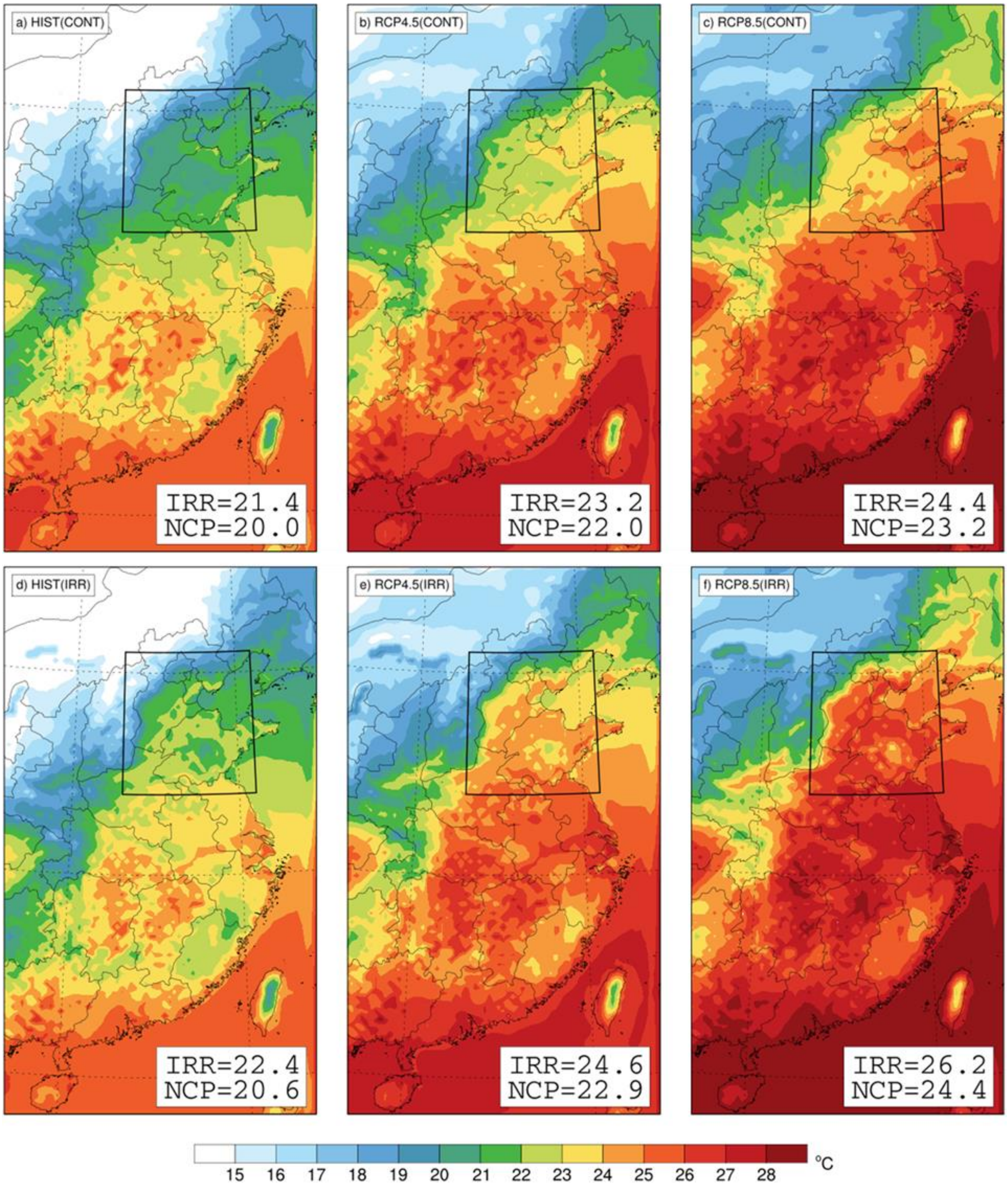

Supplementary Figure 4. Spatial distribution of ensemble mean of July-August-September 30-year climatology of TWmax (°C) for irrigation activity and each GHG scenario: historical without irrigation activity (a), RCP 4.5 without irrigation activity (b), RCP8.5 without irrigation activity (c), historical with irrigation activity (d), RCP 4.5 with irrigation activity (e) and RCP8.5 with irrigation activity (f). Averages for irrigated region (IRR) and North China Plain (box in plot, NCP) are indicated in each plot. Extent of irrigated area is shown in Supplementary Figure 1. TWmax is maximum daily value from 6-hour running average for each day (bias correction described in Method). The figure was created using the NCAR Command Language (https://www.ncl.ucar.edu)

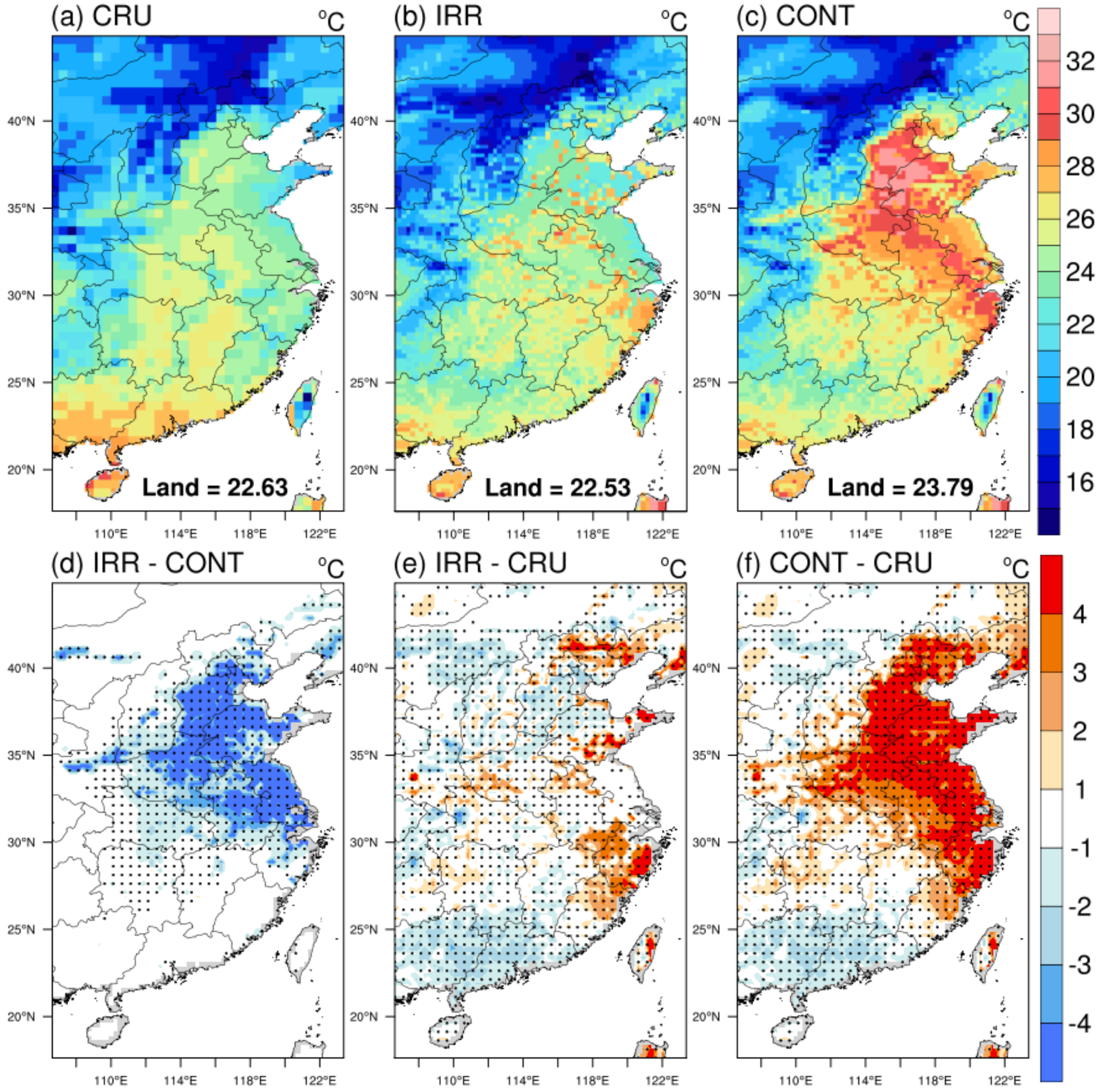

Supplementary Figure 5. Spatial distribution of May-June-July 30-year (1982–2011) climatology of surface temperature (°C) for CRU and MRCMs driven by ERA-interim. (a) CRU; (b) MRCM simulation with irrigation (IRR); (c) MRCM simulations without irrigation (CONT); (d) difference between IRR and CONT; (e) difference between IRR and CRU; (f) difference between CONT and CRU. MRCMs are interpolated to the coarser CRU grid. Stippling in (d-f) indicates regions where the differences are statistically significant at the 5% level as determined by a two-sided Student's t-test. The figure was created using the NCAR Command Language (https://www.ncl.ucar.edu)

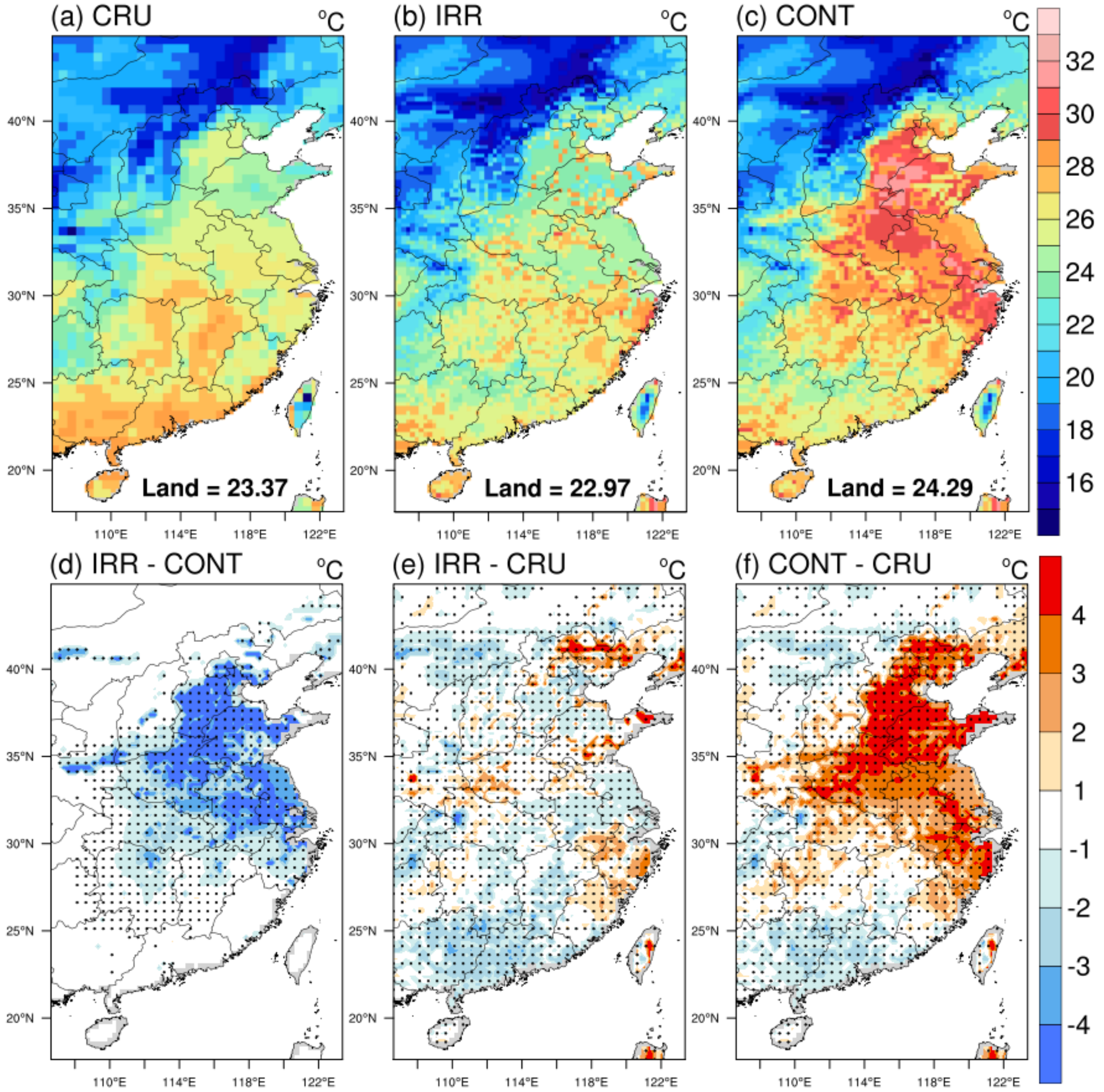

Supplementary Figure 6. Spatial distribution of July-August-September 30-year (1982–2011) climatology of surface temperature (°C) for CRU and MRCMs driven by ERA-interim. (a) CRU; (b) MRCM simulation with irrigation (IRR); (c) MRCM simulations without irrigation (CONT); (d) difference between IRR and CONT; (e) difference between IRR and CRU; (f) difference between CONT and CRU. MRCMs are interpolated to the coarser CRU grid. Stippling in (d-f) indicates regions where the differences are statistically significant at the 5% level as determined by a two-sided Student's t-test. The figure was created using the NCAR Command Language (https://www.ncl.ucar.edu)

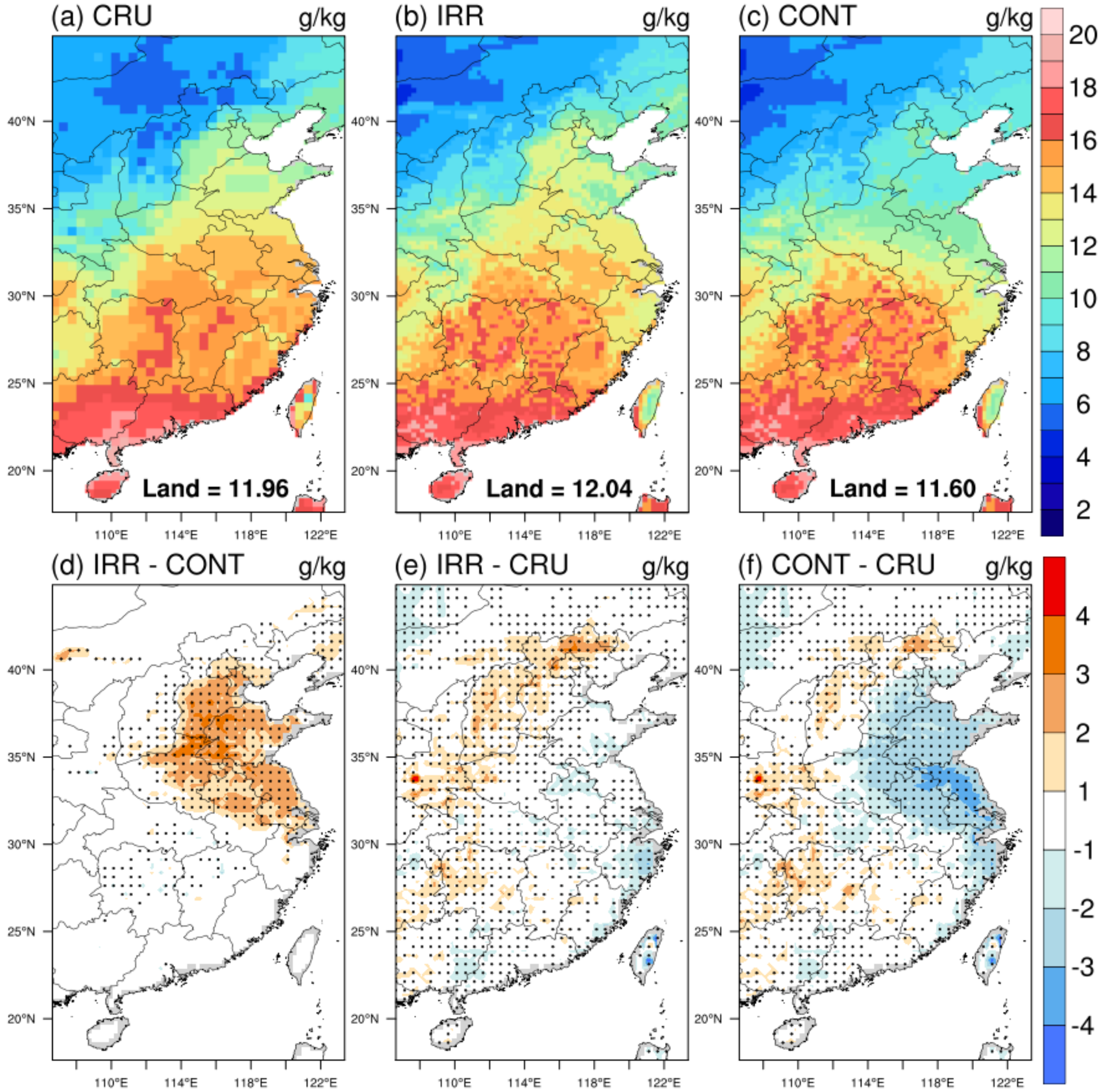

Supplementary Figure 7. Spatial distribution of May-June-July 30-year (1982–2011) climatology of specific humidity (g/kg) for CRU and MRCMs driven by ERA-interim. (a) CRU; (b) MRCM simulation with irrigation (IRR); (c) MRCM simulations without irrigation (CONT); (d) difference between IRR and CONT; (e) difference between IRR and CRU; (f) difference between CONT and CRU. MRCMs are interpolated to the coarser CRU grid. Stippling in (d-f) indicates regions where the differences are statistically significant at the 5% level as determined by a two-sided Student's t-test. The figure was created using the NCAR Command Language (https://www.ncl.ucar.edu)

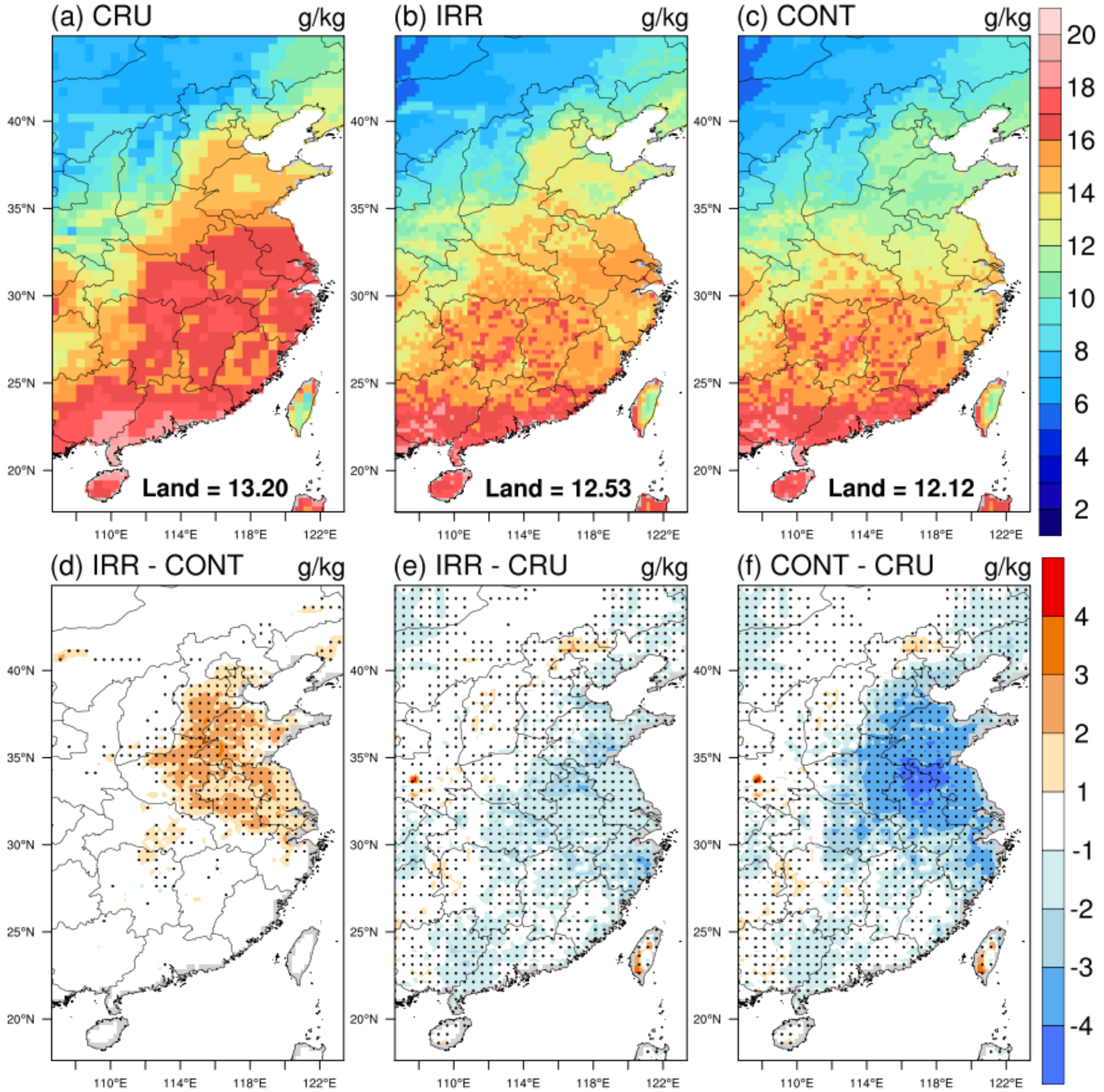

Supplementary Figure 8. Spatial distribution of July-August-September 30-year (1982–2011) climatology of specific humidity (g/kg) for CRU and MRCMs driven by ERA-interim. (a) CRU; (b) MRCM simulation with irrigation (IRR); (c) MRCM simulations without irrigation (CONT); (d) difference between IRR and CONT; (e) difference between IRR and CRU; (f) difference between CONT and CRU. MRCMs are interpolated to the coarser CRU grid. Stippling in (d-f) indicates regions where the differences are statistically significant at the 5% level as determined by a two-sided Student's t-test. The figure was created using the NCAR Command Language (https://www.ncl.ucar.edu)

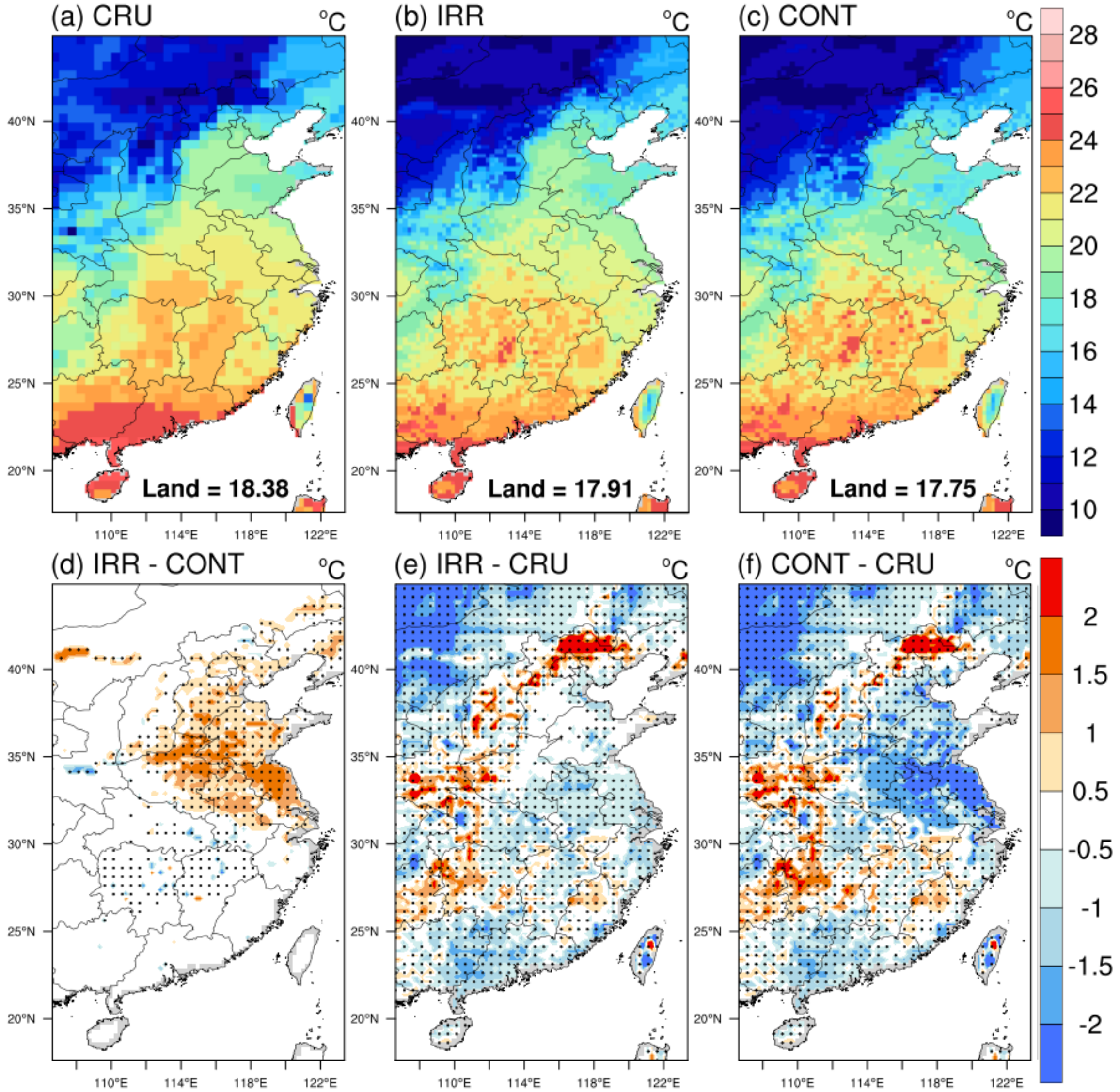

Supplementary Figure 9. Spatial distribution of May-June-July 30-year (1982–2011) climatology of wet-bulb temperature (°C) for CRU and MRCMs driven by ERA-interim. (a) CRU; (b) MRCM simulation with irrigation (IRR); (c) MRCM simulations without irrigation (CONT); (d) difference between IRR and CONT; (e) difference between IRR and CRU; (f) difference between CONT and CRU. MRCMs are interpolated to the coarser CRU grid. Stippling in (d-f) indicates regions where the differences are statistically significant at the 5% level as determined by a two-sided Student's t-test. The figure was created using the NCAR Command Language (https://www.ncl.ucar.edu)

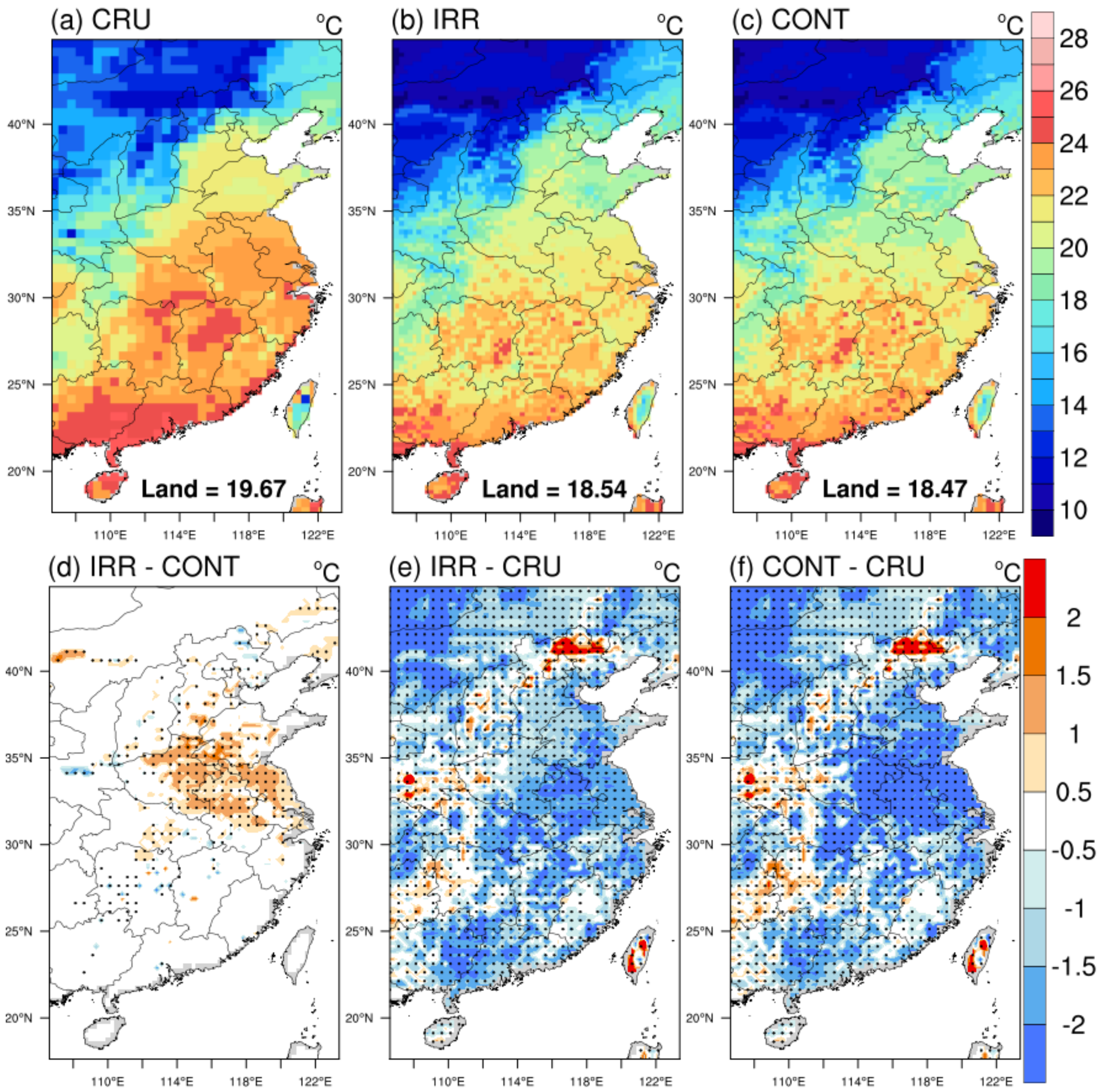

Supplementary Figure 10. Spatial distribution of July-August-September 30-year (1982–2011) climatology of wet-bulb temperature (°C) for CRU and MRCMs driven by ERA-interim. (a) CRU; (b) MRCM simulation with irrigation (IRR); (c) MRCM simulations without irrigation (CONT); (d) difference between IRR and CONT; (e) difference between IRR and CRU; (f) difference between CONT and CRU. MRCMs are interpolated to the coarser CRU grid. Stippling in (d-f) indicates regions where the differences are statistically significant at the 5% level as determined by a two-sided Student's t-test. The figure was created using the NCAR Command Language (https://www.ncl.ucar.edu)
